# Temporal and geographical variations in diagnostic imaging in Norway

**DOI:** 10.1186/s12913-024-10869-5

**Published:** 2024-04-12

**Authors:** Bjørn Morten Hofmann, Ingrid Øfsti Brandsaeter, Eivind Richter Andersen, Jan Porthun, Elin Kjelle

**Affiliations:** 1 Department of Health Sciences Gjøvik, Norwegian University of Science and Technology (NTNU), NTNU Gjøvik, PO Box 191, 2802 Gjøvik, Norway; 2 Centre for Medical Ethics, University of Oslo, PO Box 1130, 0318 Blindern, Oslo, Norway

**Keywords:** Imaging, MRI, CT, Ultrasound, Radiograph, Variation, Safety, Quality, Efficiency

## Abstract

**Background:**

Unwarranted temporal and geographical variations are acknowledged as a profound problem for equal access and justice in the provision of health services. Even more, they challenge the quality, safety, and efficiency of such services. This is highly relevant for imaging services.

**Objective:**

To analyse the temporal and geographical variation in the number of diagnostic images in Norway from 2013 to 2021.

**Methods:**

Data on outpatient imaging provided by the Norwegian Health Economics Administration (HELFO) and inpatient data afforded by fourteen hospital trusts and hospitals in Norway. Data include the total number of imaging examinations according to the Norwegian Classification of Radiological Procedures (NCRP). Analyses were performed with descriptive statistics.

**Results:**

More than 37 million examinations were performed in Norway during 2013–2021 giving an average of 4.2 million examinations per year. In 2021 there was performed and average of 0.8 examinations per person and 2.2 examinations per person for the age group > 80. There was a 9% increase in the total number of examinations from 2013 to 2015 and a small and stable decrease of 0.5% per year from 2015 to 2021 (with the exception of 2020 due to the pandemic). On average 71% of all examinations were outpatient examinations and 32% were conducted at private imaging centres. There were substantial variations between the health regions, with Region South-East having 53.1% more examinations per inhabitant than Region West. The geographical variation was even more outspoken when comparing catchment areas, where Oslo University Hospital Trust had twice as many examinations per inhabitant than Finnmark Hospital Trust.

**Conclusion:**

As the population in Norway is homogeneous it is difficult to attribute the variations to socio-economic or demographic factors. Unwarranted and supply-sensitive variations are challenging for healthcare systems where equal access and justice traditionally are core values.

**Supplementary Information:**

The online version contains supplementary material available at 10.1186/s12913-024-10869-5.

## Introduction

Unwarranted temporal and geographical variations are acknowledged as a profound problem for equal access and justice in the provision of health services [1–6]. Moreover, it challenges the quality, safety, and efficiency of such services.

In diagnostic imaging, as in many other fields in medicine, temporal and geographical variations are well documented internationally [7–15] as well as in Norway [12, 16–20]. For example, OECD statistics has documented a substantial temporal increase in imaging as well as large variations between countries. While USA had 255 CT and 108 MRI examinations per 1000 inhabitants, Romania had 58 and 21 examinations, respectively in 2021 (https://stats.oecd.org/).

However, studies of variations are mainly for specific imaging procedures [13, 21–25], certain services [17], specific organizations [14], or by specific sampling methods [12, 26]. Thus, in order to study, plan, and improve imaging services for entire healthcare jurisdictions, we need more knowledge of long-term trends and variations for imaging in whole countries. Moreover, variations in the health services of a demographically very homogenous country are interesting as it can point to important factors influencing potentially unwarranted variations.

Accordingly, the objective of this study was to analyse the temporal and geographical variation in the number of diagnostic images in Norway from 2013 to 2021.

## Methods

### Setting

In the Norwegian healthcare system imaging services is organized as part of the specialist healthcare system, which is organized in four Regional Health Authorities (RHAs): Region South-East, Region West, Central Region, and Region North. Within each RHA there are several health trusts (HT) including one or more hospitals [27]. The capital, Oslo, is included in Region South- East which holds a larger population than the other RHAs and covers about 57% of the Norwegian population. Moreover, this RHA also hosts two hospitals with national tertiary specialist health service responsibilities in for instants oncology, paediatrics, and transplantations.

There are 19 public health trusts with one or more imaging departments and 24 private imaging centres partly commissioned by public health services and partly providing access to outpatient services through out-of-pocket payment or private health insurance policies [28]. Additionally, some hospitals and institutions run by ideal organizations have radiological facilities. Nearly all the private imaging centres have agreements with the health care trusts and receive the same reimbursement independent of where examinations are performed.

Norway has universal health coverage which is funded through a general tax system and minor cost-sharing payments/co-payments [28]. The co-payment rate for radiology was €25 per examination in 2023. Co-payments have a ceiling of €271 in total for many types of care. Beyond this ceiling no co-payment is needed for the rest of the calendar year, neither for imaging nor for other public services [29].

#### Material

Outpatient data was collected centrally from the Norwegian Health Economics Administration (HELFO). Inpatient data was collected directly from hospital trusts covering 68% of the Norwegian population. The data include examination codes in the Norwegian Classification of Radiological Procedures (NCRP) system, name of procedure, modality, hospital/imaging centre, patients’ age, and sex, and in- or outpatient status.

Data on the Norwegian population in the various geographical areas was provided by Statistics Norway for each year and age group. The data on the number of imaging machines is provided by the Norwegian Radiation and Nuclear Safety Authority (DSA).

As it takes time for the providers to ascertain the data, and to request, receive, and standardize the data for analyses, the newest data that can be presented is from 2021.

The following modalities are included in this study: conventional radiology including fluoroscopy (CR), computed tomography (CT), magnetic resonance imaging (MRI), ultrasound (US), and nuclear medicine (NM).

#### Methods

Statistical analyses were performed with SPSS Statistics, version 28 (IBM Corp.) and Microsoft Excel 2016 was used for descriptive statistics. Since this study was not designed as a confirmatory study, but to identify patterns, it was not necessary to make an adjustment for tests for multiplicity. The significance tests used therefore have a descriptive character.

Extrapolation to estimate the total number of inpatients was based on population characteristics and outpatient imaging use as hospitals covering 32% of the population did not provide inpatient data.

To compare the differences between the catchment areas we used the Chi-Square Test. A p-value < 0.05 was considered statistically significant in all analyses.

RHAs, health trusts (HTs), and special (national) units were used as geographical units in accordance to Norwegian health authorities’ organization [30].

Only ultrasound examinations performed at radiological units (and not at other departments by other specialties) are included in this study.

## Results

**Fig. 1:**
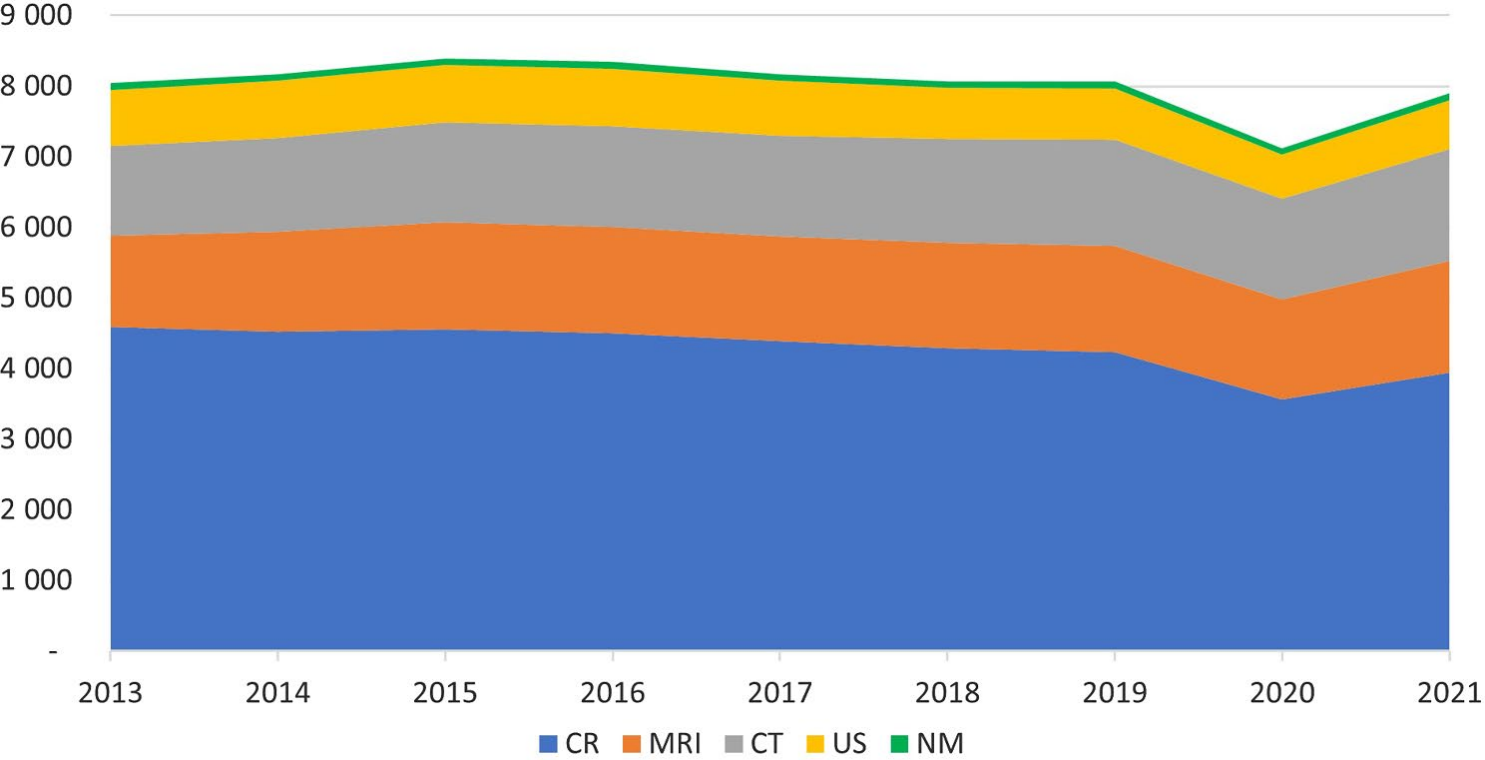
Temporal variations in imaging for the years 2013–2021 per 10,000 inhabitants for main modalities, conventional radiology including fluoroscopy (CR), magnetic resonance imaging (MRI), computed tomography (CT), ultrasound (US), and nuclear medicine (NM)

For the period 2013–2021 a total of 37,871,276 examinations were performed giving an average of 4,207,920 examinations per year. 54% of the examinations where on women and 46% were on men, and the proportion was stable in the study period. In 2021 a total average of 0.79 examination per inhabitant per year were performed. For the age group > 80 there were performed 2.15 examinations per person in 2021. The temporal variation of the total number of examinations per 10,000 inhabitants is shown in Fig. 1.

**Fig. 2:**
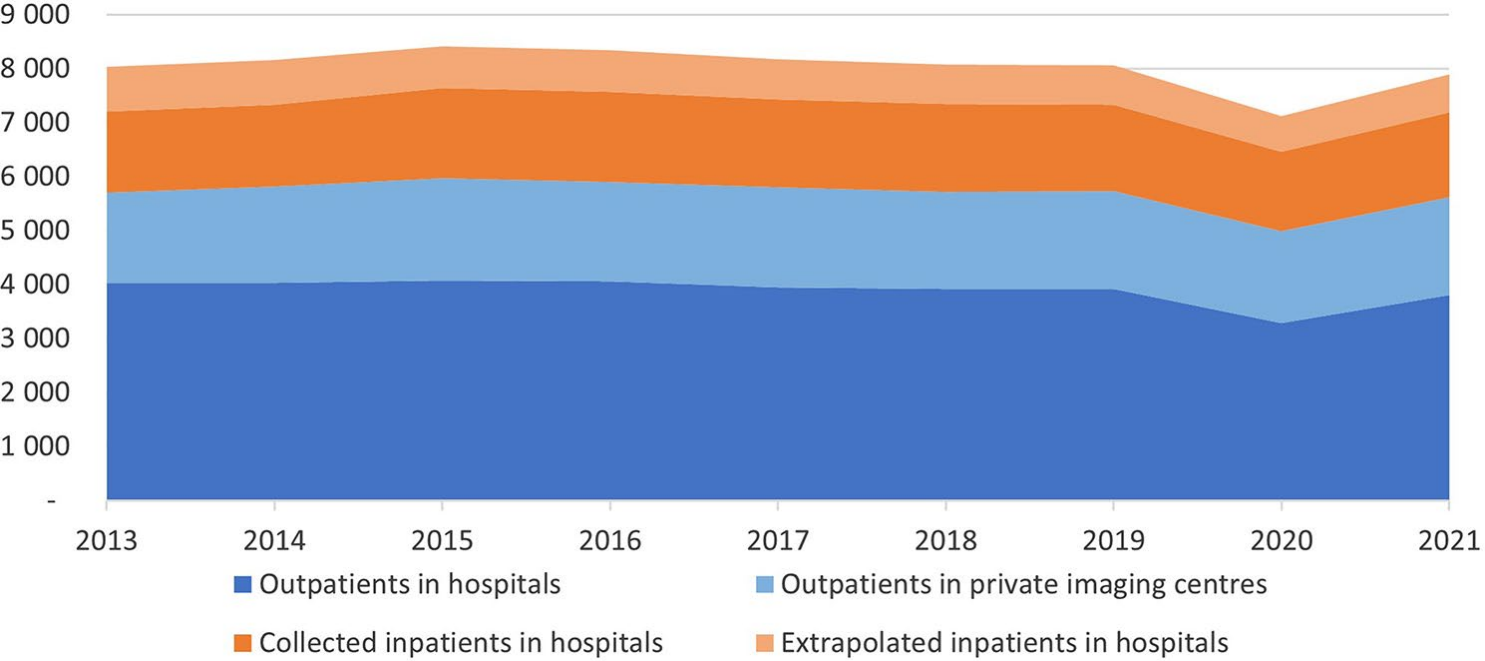
Temporal variation per 10,000 inhabitants, distribution between in- and outpatient and extrapolated inpatient data. Private imaging centres only have outpatient imaging

Annually, on average, 71% of the imaging conducted in Norway were outpatient examinations whereof 32% were conducted at private imaging centres. 22% of all imaging was conducted as outpatient imaging at private imaging centres. Figure 2 shows the temporal variation for public and private imaging centres as well as for in-and outpatient imaging from 2013 to 2021.

On average, the number of examinations were reduced with 11.0% during the pandemic year 2020 compared to the average of the (stable) years from 2015 to 2019. The largest reduction was identified in the number of CR (19.9%) and US (19.3%) in the Region South-East. Interestingly, there was an increase in the number of examinations in 2020 in the number of some examinations, especially for NM in Region North (12.8%) and Region West (7.7%) compared to the average.

**Fig. 3:**
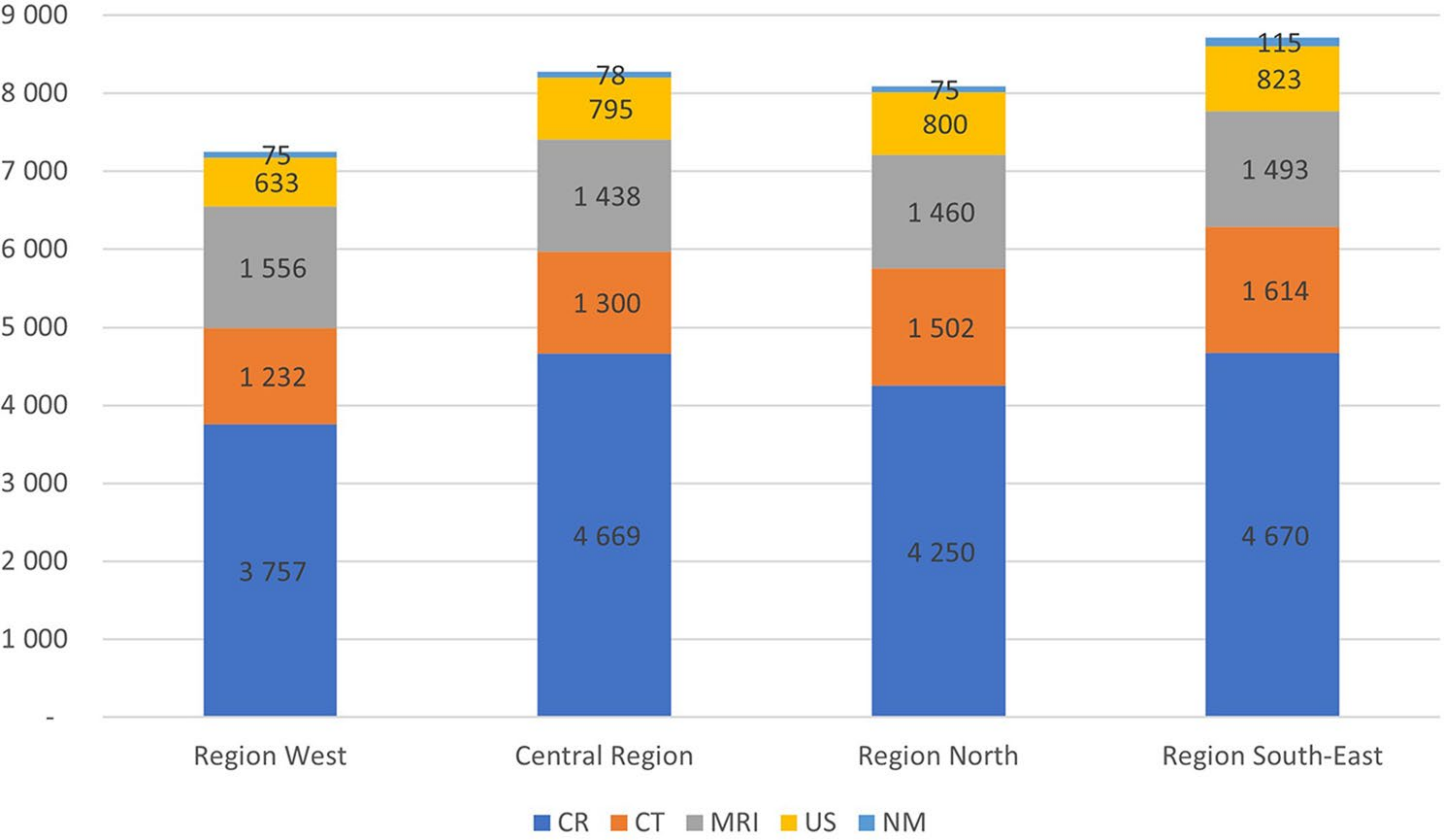
Average regional differences in imaging for the years 2013–2021 per 10,000 inhabitants for conventional radiology including fluoroscopy (CR), computed tomography (CT), magnetic resonance imaging (MRI), ultrasound (US), and nuclear medicine (NM)

Figure 3 shows the average regional differences in imaging for the years 2013–2021 per 10,000 inhabitants for main modalities. Overall, Region South-East had 19.4% more examinations per 10,000 inhabitants than Region West. The biggest difference is in the number of NMs where Region South-East had 53.1% more examinations than Region West. This is probably due to centralized national NM services in Oslo. Nonetheless, Region South-East had 30.1% more CT examinations than Region West. On the other hand, Region West had 9.1% more MRIs than the Central Region on average for the years 2013 to 2021.

**Fig. 4:**
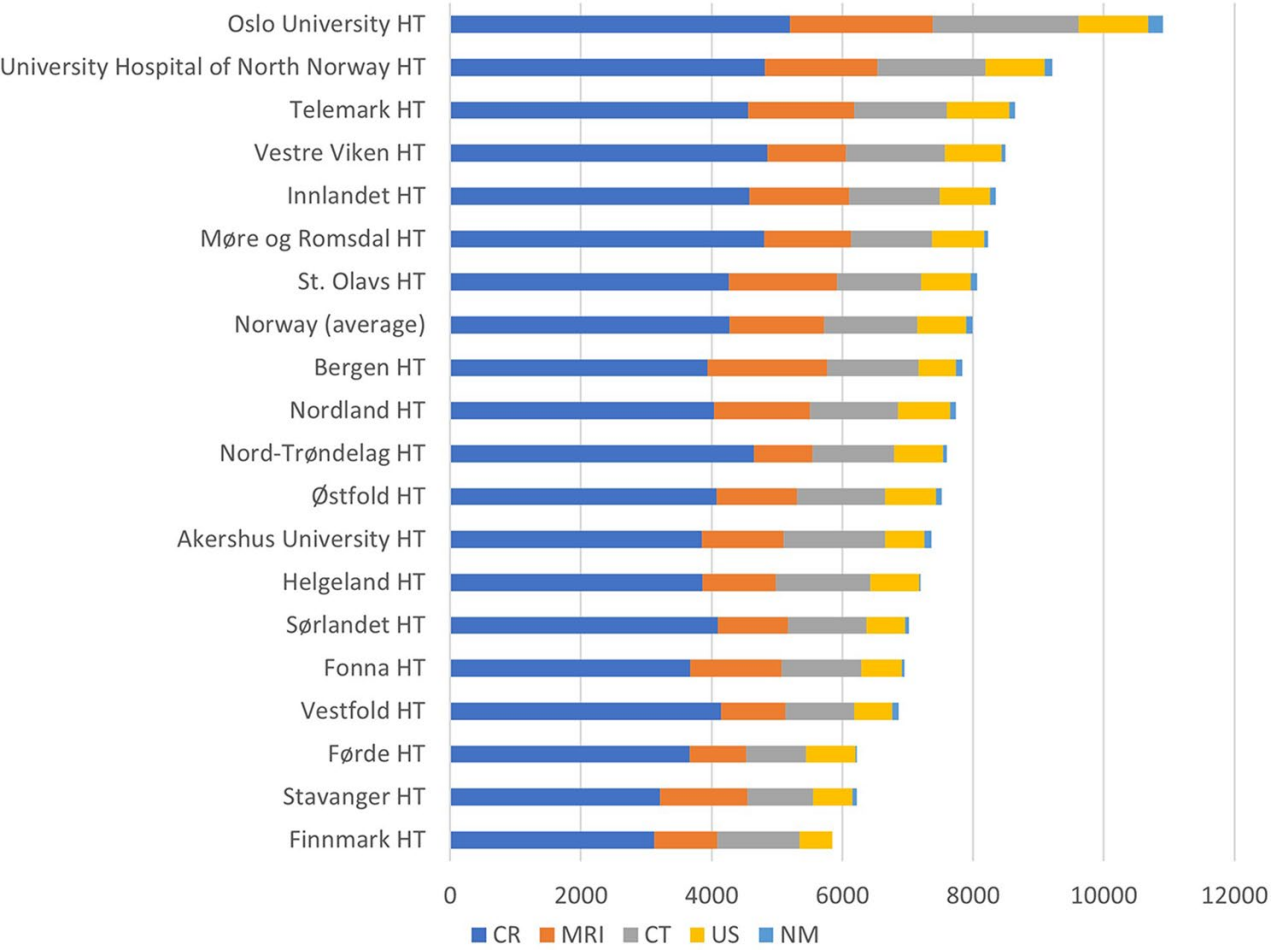
Average geographical variations in imaging for the years 2013–2021 per 10,000 inhabitants for the hospital trusts catchment areas in Norway

There are also substantial differences in imaging between the catchment areas in Norway as shown in Fig. 4. There were on average almost twice as many examinations per inhabitant in Oslo than in Finnmark (96.2% more) for the years 2013 to 2021. This difference between the catchment areas is statistically significant (*p* = 0.043). It is also interesting to notice that there are substantial differences between hospital trusts within the same region.

**Fig. 5:**
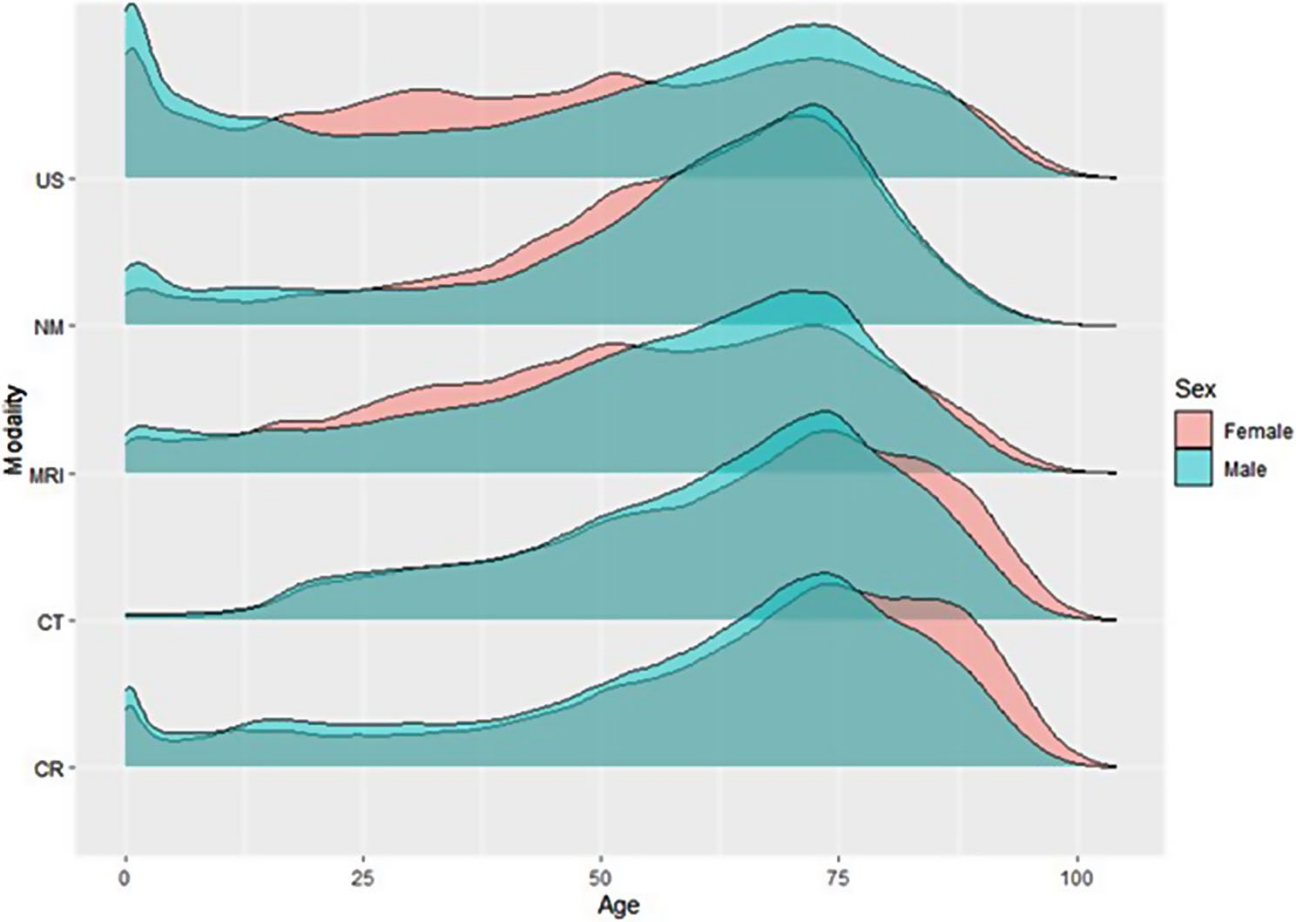
Density plot showing age distribution for imaging in public hospitals by sex and modality

Figure 5 shows the distribution of examinations for female and male for the various age groups. Clearly the age group between 50 and 90 receive most imaging per person. While males dominate below the age of 78, female dominate above (due to longer average lives).

The age distributions between female and male patients for the various regions are very similar. Imaging increases equally until age of 50. Between 50 and 80 years, male dominate whereafter female clearly dominate. Density plot for age distribution for all regions is presented in Supplement Figure S1 and for all modalities is presented in Figure S2.

As only hospitals provided year-specific age information on the persons examined, comparison with private providers could not be presented in density plots. However, histograms for age distribution, based on age groups, including private providers are presented in Figures S3 and S4.

**Fig. 6:**
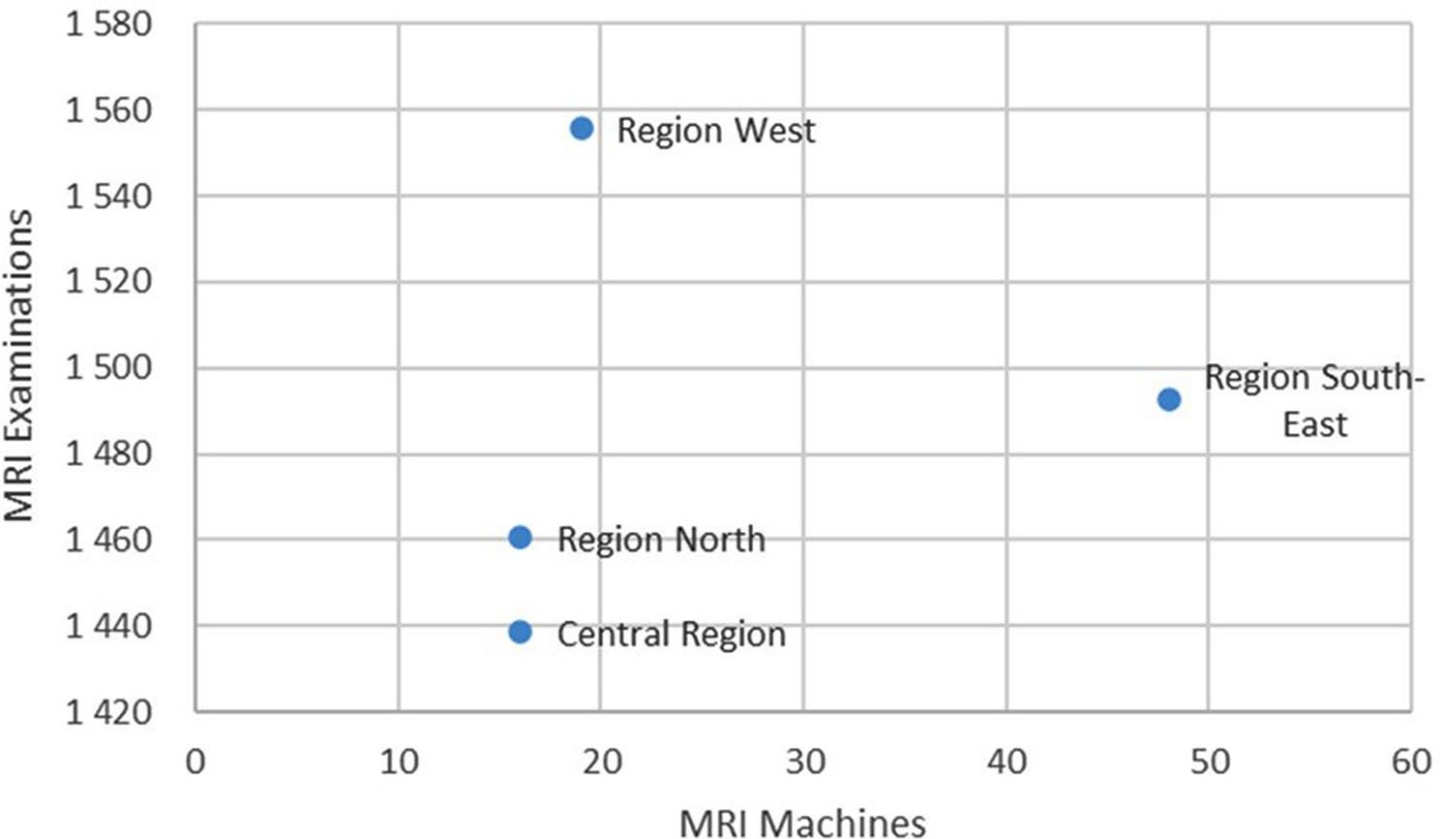
Scatter plot of the number of MRI examinations per 10,000 inhabitants versus number of registered MRI machines for the four RHAs

While the regions West, North, and the Central Region have comparable number of MRI machines, Region West makes more MRI examinations per 10,000 inhabitants per year than the other regions. Region West also makes more MRI examinations per 10,000 inhabitants than Region South-East who have more than twice as many MRI machines, as illustrated in Fig. 6.

## Discussion

More than 37 million examinations were performed in Norway during 2013–2021 giving an average of 4.2 million examinations per year. In 2021 there was performed an average of 0.79 examinations per person in Norway and 2.15 examinations per person for the age group > 80. There was a 9% increase in the total number of examinations in Norway from 2013 to 2015 and a small and stable decrease of 0.5% per year from 2015 to 2021 (with the exception of 2020 due to the pandemic). On average, 71% of all examinations in Norway were outpatient examinations, and of these, 32% were conducted at private imaging centres. While the temporal variations are moderate, there are substantial geographical variations. Region South-East performs most examinations, while Region North and Central Region are very similar, and Region West has fewer examinations per inhabitant per year. There are marked differences in number of examinations for the various age groups and between female and male patients. There are also differences in examinations for the various age groups with respect to modalities and regions. To our knowledge this is the first study that presents temporal and geographical variations for a whole country for a period as long as nine years.

According to Statistics Norway, Norway is a very homogenous country with small differences in morbidity and mortality as well as socioeconomic status. Hence, the geographical variations must be due to different professional profile or preferences (e.g., with respect to modalities), access to facilities, and urbanity. Region South-East includes Oslo (Fig. 3), is more urban, and has some more national functions involving imaging than the other regions, which could explain some of the extra examinations. However, some of these specialties do not generate a great number of images. Moreover, although access to imaging facilities is known to play an important role [31], this cannot explain that Region North has a higher utilization of imaging services than Region West.

For MRI examinations, the number of machines in the Central Region, Region North, and Region West are comparable. However, the remoteness, harsh climate, and travel alternatives yield specific challenges in providing healthcare in Northern Norway [32]. Consequently, a significant portion of the population in Region North have more difficult access to imaging services compared to people living in the southern parts of the country. It is therefore puzzling that Region North has a higher utilization rate on US, CT, and MRI than the Central Region. One reason could be that inhabitants in the remote areas are used to travel far (in harsh climate) to access services, and therefore experience to have good access to imaging facilities. There may also be local compensatory mechanisms at play. For example, people living in the Sami speaking areas have significantly less CT and US exams, but more MR exams than in other comparable areas [33].

Conversely, Region West, which has a lower overall utilization rate (but a higher MRI utilization rate), include two large cities with about 290,000 and 150,000 inhabitants and covers a total population of 1.14 million inhabitants. Here, communication and access to health services are at least as good as in the Central Region and Region North. The overall lower utilization rates in Region West can therefore not be explained by access to services.

Differences in research activities may also explain the geographical variations. However, the research activities are comparable between the regions. While there is some patient migration to Oslo [26], this cannot explain the whole difference in utilization. On the other hand, some parts of Region South-East have more immigrants and persons with low socioeconomic status, who tend to use primary health care services less frequently [34]. For imaging services, we have no data.

Hence, the discussed differences can hardly explicate the lower use of imaging services in Region West and the higher use in Region South-East. Potential explanations, to investigate in further studies, are that there are differences in expectations and demands between people living across regions. Whilst supply-sensitive services and organizational culture is documented to be determinant for overuse and low-value care [35], it is specifically documented that both patient expectations, and local culture and procedures are drivers for unnecessary imaging utilization in Norway [36]. Furthermore, the great variation in referral practice documented [37] might yield variation in imaging utilization. Consequently, the results correspond well with the findings with the Office of the Auditor General of Norway [38], with national analyses of outpatient data (https://www.skde.no/helseatlas/v2/radiologi/), and with studies of specific examinations [17, 39].

The results also agree with international studies investigating geographical variations showing substantial variations for the use of CT, MRI, and CR in several countries and contexts [7, 11, 13, 14, 15, 23, 40, 41]. They are also in line with the statistics of the OECD on CT and MRI (https://stats.oecd.org/). As can be expected in a country with universal coverage and with strong equity-aspirations, the variations are smaller than in countries with more emphasis on private healthcare. However, despite homogeneity, the variations are puzzling. The temporal reductions in examinations in 2020 are in accordance with other studies showing a substantial reduction (50%) in a short period of time during the pandemic [42].

Much of the variation in the private sector may be due to differences in access to private services, which is much higher in urban areas like Oslo than in rural catchment areas, such as in Finnmark. This in turn can indicate that at least part of the variation demonstrated in this study can be explained by accessibility of service, since some will resort to private services (this is especially the case for musculoskeletal examinations). However, the population density is higher in these areas and there are reasons to believe that more people pay out of pocket here (and thus are not included in this study).

One potential explanation for access being a key factor of utilization is long wait times in the public system for very many diagnostic imaging examinations (up to 52 weeks), while wait times are much shorter in the private services (up to 12 weeks). This accounts for some imaging migration and implicit division of labor, corresponding to a recent study from Norway indicating that people are willing to travel to receive imaging services [37].

In the scatter plot (Fig. 6) Region South-East appears to be an outlier displaying a higher number of MRI machines without a proportional increase in examinations per 10,000 inhabitants. This does not necessarily imply that Region South-East conducts fewer examinations per MRI machine compared to other regions. The larger population size in the region could account for the seemingly lower examination rates per machine. Accordingly, further research should scrutinize the actual accessibility of imaging equipment in Norway.

One strength of this study compared to other studies is that we were able to include inpatient imaging. Although we were not able to acquire a full data set, we obtained data for 68% of all inpatients and using population data and outpatient imaging use to extrapolate gives a good estimate of the total number of images in Norway.

While we have investigated outpatient examinations paid by the public service, some examinations were paid either fully by the patient or by health insurance. For example, 8.7% of examinations performed by the private provider Unilabs in 2017 were paid either fully by the patient or by health insurance (or 50 925 examinations out of a total 580 097) [43]. In general, about 10% of imaging in Norway is paid out of pocket or by private health insurance [28]. The rest of the examinations performed in this organization were paid through HELFO. This corresponds also to other private providers [43].

There may be many mechanisms behind temporal and geographical variations for inpatient and outpatient examinations. From previous studies we know that access and technological development (of the modalities) have been considered to be a driving force [31]. However, the overall reduction in more recent years indicates that there be a saturation effect flattening the curve in imaging.

This study has applied a 12 month sample time which evens out a wide range of shorter temporal variations. Additionally, local variations and temporal shifts in coding practices can have occurred and can provide some distortions.

As this study reports imaging from a specific country, the results cannot be extrapolated or generalized to other countries. Nonetheless, the results are of interest for comparison with other countries and other kinds of health care systems. Norway has one of the highest consumption of medical imaging [44], and can be of great interest to other countries, especially those with healthcare systems that are similar to the Nordic countries. Moreover, Norway is a very homogenous country where equity is a key principle in the provision of healthcare services. Variations in such settings are important to study as supply-driven variations. Additionally, health services in Western European countries are extensively inspired by the British NHS but display considerable variations. It is important to document and analyze similarities and differences between the systems. In the case of medical imaging, there are few complete sources of information. Data from OECD are incomplete and haunted by methodological differences in data procurement and analyses between countries. Hence, studies of this kind are important for future comparisons.

Moreover, documented variations do not say anything about the right number of examinations in an area. However, unexplained variations indicate that over- or underuse are relevant hypotheses to test. Moreover, great variation may indicate lack of adherence to appropriateness criteria [45–52] and the use of low-value examinations. Further studies are needed to investigate this.

## Conclusion

While the temporal variation in the number of imaging procedures per year per inhabitant in Norway are moderate for the years from 2013 to 2021, there were substantial geographical variations between the health regions. Region South-East had 53.1% more examinations per inhabitant than Region West. The geographical variation was even more outspoken when comparing catchment areas, where Oslo University Hospital Trust had twice as many examinations per inhabitant than Finnmark Hospital Trust. The homogeneous population in Norway makes it difficult to attribute the variations to socio-economic or demographic factors, indicating that there can be unwarranted differences, challenging the traditionally strong values of equity and justice in Norway.

### Electronic supplementary material

Below is the link to the electronic supplementary material.

Supplementary Material 1

## Data Availability

The datasets generated and analysed during the current study are not publicly available due regulation by the REC. However, aggregated data are available from the corresponding author on reasonable request.

## Declarations

All methods were carried out in accordance with relevant guidelines and regulations.

## Abbreviations

AHUS: Akershus University Hospital Health Trust
CR: Conventional x-ray
CT: Computed tomography
MRI: Magnetic resonance imaging
NCRP: Norwegian Classification of Radiological Procedures
NM: Nuclear medicine
OECD: Organisation for Economic Co-operation and Development
US: Ultrasound
UNN: University Hospital of Northern Norway
VVHF: Vestre Viken Health Trust
