# Supplementary material for: Temporal and geographical variations in diagnostic imaging in Norway
Source: BMC Health Serv Res. 2024 Apr 12;24:463. doi: 10.1186/s12913-024-10869-5 (PMC11015609; doi:10.1186/s12913-024-10869-5)
Supplement: Supplementary file 1 — Supplementary Material 1 [file 12913_2024_10869_MOESM1_ESM.docx]

# Supplement

**
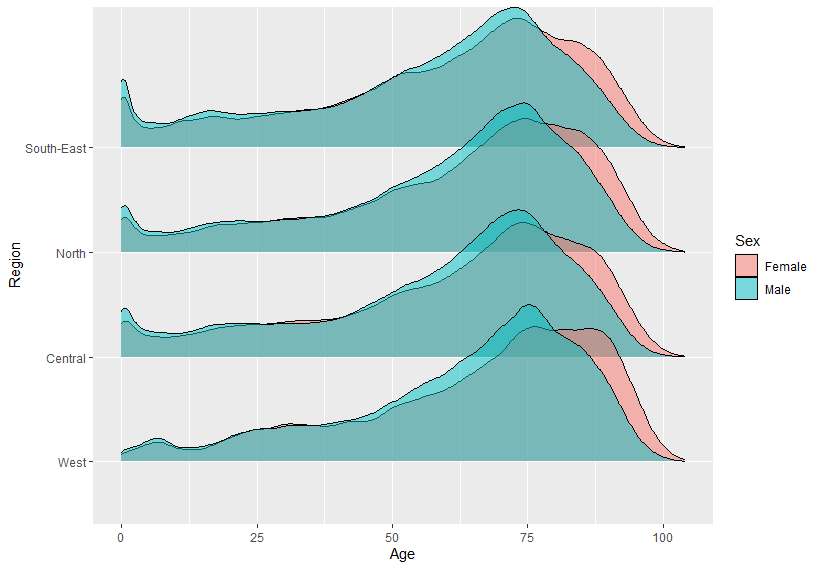
**

**Figure S1** Density plot showing age distribution only for imaging in public hospitals by sex and region.

**
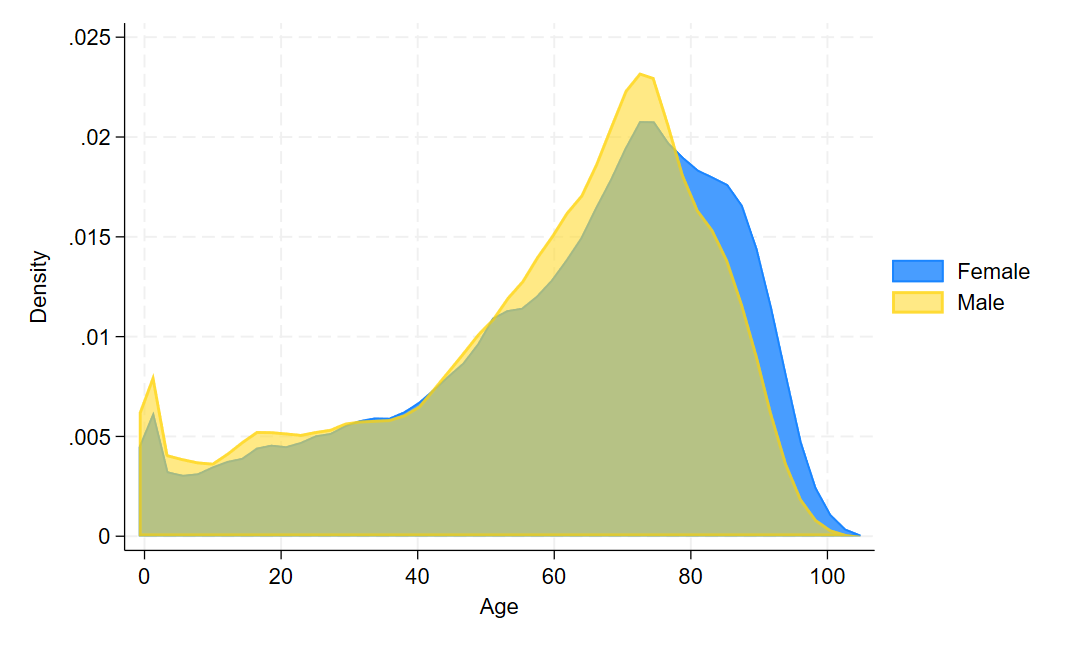
**

**Figure S2** Density plot showing age distribution for imaging performed in hospitals by sex and all primary modalities combined.


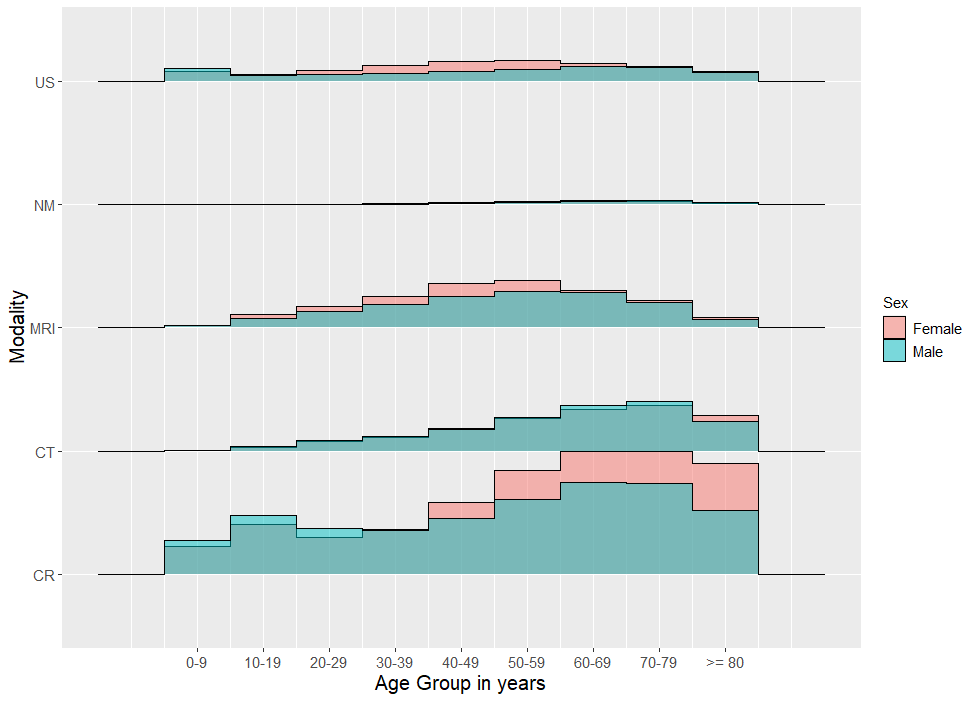


**Figure S3** Histogram showing age-group distribution by sex and primary modalities for hospitals and private images center together.


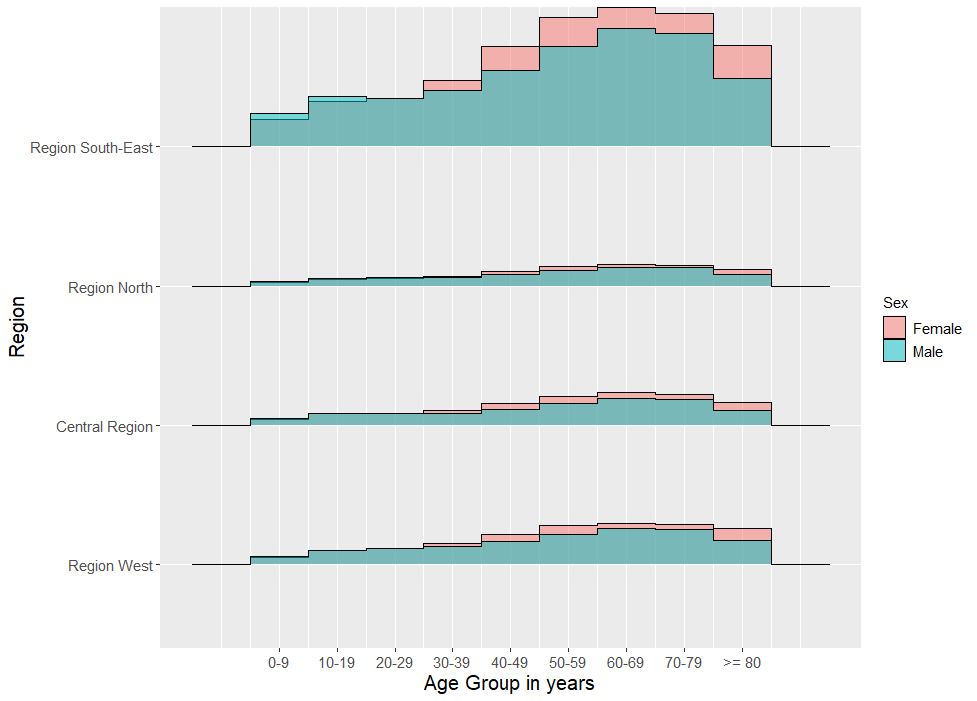


**Figure S4** Histogram showing age-group distribution by sex and regions for hospitals and private images center together.

Hospitals provided year-specific age information on the persons examined, while the private imaging centres provided age-grouped data. Therefore, we only present age distributions from hospitals (figures 5, S1, and S2). However, histograms for age distribution, based on age groups, summarise the first ten years of life (figures S3 and S4). The histograms therefore camouflage that there are more examinations in the first year of life than in the years after, as can be seen from the figures with exact ages (figures 5 and S1).
